# Chemotherapeutic Compounds Targeting the DNA Double-Strand Break Repair Pathways: The Good, the Bad, and the Promising

**DOI:** 10.3389/fonc.2014.00086

**Published:** 2014-04-22

**Authors:** Christian Jekimovs, Emma Bolderson, Amila Suraweera, Mark Adams, Kenneth J. O’Byrne, Derek J. Richard

**Affiliations:** ^1^Cancer and Ageing Research Program, Institute of Health and Biomedical Innovation, Queensland University of Technology, Brisbane, QLD, Australia

**Keywords:** DNA damage repair, chemotherapeutic compounds, DNA damage response, cancer, radiosensitize, radioprotective, DNA double-strand break

## Abstract

The repair of DNA double-strand breaks (DSBs) is a critical cellular mechanism that exists to ensure genomic stability. DNA DSBs are the most deleterious type of insult to a cell’s genetic material and can lead to genomic instability, apoptosis, or senescence. Incorrectly repaired DNA DSBs have the potential to produce chromosomal translocations and genomic instability, potentially leading to cancer. The prevalence of DNA DSBs in cancer due to unregulated growth and errors in repair opens up a potential therapeutic window in the treatment of cancers. The cellular response to DNA DSBs is comprised of two pathways to ensure DNA breaks are repaired: homologous recombination and non-homologous end joining. Identifying chemotherapeutic compounds targeting proteins involved in these DNA repair pathways has shown promise as a cancer therapy for patients, either as a monotherapy or in combination with genotoxic drugs. From the beginning, there have been a number of chemotherapeutic compounds that have yielded successful responses in the clinic, a number that have failed (CGK-733 and iniparib), and a number of promising targets for future studies identified. This review looks in detail at how the cell responds to these DNA DSBs and investigates the chemotherapeutic avenues that have been and are currently being explored to target this repair process.

## Introduction

Genomic stability at a cellular level requires precise, tightly coordinated pathways to detect DNA damage and either repair the damage or, if the damage is too great, ensure the cell dies via apoptosis or enters senescence. Organisms have evolved complex DNA damage response (DDR) pathways to respond to insults to the DNA either from endogenous (cellular metabolic pathways, reactive oxygen species, and errors in DNA replication) or exogenous sources [environmental factors including ionizing radiation (IR) and ultra violet radiation].

Cellular DNA damage that is not repaired correctly can lead to genomic instability, apoptosis, or senescence, which can greatly affect the organism’s development and aging process and in addition can predispose the organism to immunodeficiency, neurological disorders, and cancer.

## DDR and Repair Pathways

Following the initial work on the DDR in yeast, investigations into the DDR in mammals have yielded a highly conserved and elaborate process. This process mainly controls DNA repair (ensuring genomic stability) and cell cycle checkpoints, however it has also been shown to be involved in circadian rhythms (1), insulin signaling (2), and telomere maintenance (3).

The DDR pathway encompasses a set of tightly coordinated processes: detection of DNA damage, a protein cascade to enhance the signal, the accumulation of repair factors at the site of damage, and physical repair of the damage. The DDR also induces cell cycle checkpoints to ensure the damaged cells do not continue dividing until the DNA damage is repaired. To ensure genomic stability, the DDR must be able to recognize all types of DNA structural alterations, including nicks, gaps, stalled replication, and double-strand breaks (DSBs).

Depending on the type of DNA lesion, there are a number of DNA repair pathways available for the cell to repair the alteration, including homologous recombination (HR) and non-homologous end joining (NHEJ) for DNA DSBs; and mismatch repair (MMR), nucleotide excision repair (NER), and base excision repair (BER) for single DNA strand damage.

Highlighting the importance of the DDR, mutations in a number of repair proteins lead to human syndromes, which include multiple cancers, immunodeficiency, and genomic instability phenotypes. Ataxia telangiectasia mutated (ATM), a protein involved in the DDR is mutated in the syndrome ataxia telangiectasia (AT) (4). AT is a cancer-prone syndrome that also includes progressive cerebellar ataxia, telangiectasia’s of the conjunctivae, and immunodeficiency. Consistent with ATM’s role in the DDR, AT patients presented a high level of sensitivity to radiation (5). Nijmegen breakage syndrome (NBS) is another syndrome where the key cause of the disease is a mutation in a protein involved in the DDR, NBS1. NBS1 is involved in the detection of DSBs as part of a complex of proteins including Mre11 and Rad50. NBS is a cancer-prone syndrome that is also characterized by progressive microcephaly, short stature, and progressive ovarian failure in females (6).

Current chemotherapeutic compounds development is largely focused in targeting proteins specific to pathways important to the development, growth, and progression of cancer. DSBs are the most deleterious lesion to cells, where unrepaired DSBs can lead to cell death and incorrectly repaired DSBs have the potential to produce chromosomal translocations and genomic instability, potentially leading to cancer. Targeting the repair proteins involved in the repair of DSBs with chemotherapeutic compounds has the potential for cancer therapies in conjunction with radiation therapy or as a monotherapy.

## Chemotherapeutic Compounds

Double-strand breaks are highly cytotoxic and this fact is exploited in conventional cancer treatment, with radiation therapy and chemotherapeutic drugs treatments generating vast amounts of DSBs. These include chemotherapeutic drugs that induce DNA cross-links or function as topoisomerase inhibitors, inducing the generation of DSB’s in all cells. However, cancer cells are much more susceptible to these drugs, as they are rapidly dividing and often have inactivated components of their DNA repair machinery and deregulated cell cycle checkpoints (7).

However, these chemotherapeutic drugs will also target normal proliferating cells that are dividing as part of their normal processes. These naturally regenerating tissues include bone marrow, gastrointestinal tract, liver, and hair follicles. The formation of secondary hematologic and solid tumors after DNA-damaging therapies is a potential issue for patients undergoing treatment (8).

A number of chemotherapeutic compounds are used in conjunction with radiotherapy or in combination with other chemotherapeutic agents to produce a synergistic effect. The use of radiosensitizing agents that increase the cytotoxic effects of radiation on cancer cells and radioprotective agents that decrease the adverse effects of radiation on normal cells (by increasing their radioresistance) is common. The use of radiosensitizing agents can greatly enhance the efficacy of radiotherapy and genotoxic drugs. Recently, chemotherapeutic compounds have been studied that may also be useful as a monotherapy, where the chemotherapeutic compound achieves what is termed as “synthetic lethality.” Synthetic lethality exploits the fact that many cancer cells acquire defects in DNA repair pathways and become dependent on a compensatory mechanism in order to survive (9, 10). Inhibition of the complementary DNA repair pathway selectively kills cancer cells that have a defect in a particular DNA repair pathway.

The safety, tolerability, pharmacokinetics, and efficacy of potential chemotherapeutic compounds have to be carefully validated before they may enter clinical trials to determine the benefits for cancer therapy. This means there is a significant delay between the initial discovery of a potential chemotherapeutic compound in the laboratory, to an actual clinical outcome for patients, however this delay ensures patient safety.

This review will focus on the pathways responsible for the repair of DSBs, namely HR and NHEJ, and the current chemotherapeutic compounds that are being investigated that target these repair pathways.

## The DDR Targets and Chemotherapeutic Compounds

DNA DSBs are considered the most cytotoxic of DNA lesions. Cells are estimated to accumulate around 50 endogenous DSBs per day, mostly induced by reactive oxygen species (11). In response to DSBs, the DDR utilizes two main pathways to repair the damage. During late S-phase and the G2 phase, cells have a sister chromatid available as a template for targeted HR, which allows for error-free repair of the DNA damage. However, DSBs that occur when there is no sister chromatid available are repaired via NHEJ, which is more error-prone than HR. NHEJ is also active in S and G2 phases of the cell cycle and remains the predominant pathway by which cells repair DSBs. In NHEJ, the two ends of the break are joined together (ligated), though this can involve resection with the consequent loss of genetic material (12). Cancer therapy agents induce DSBs including IR and topoisomerase II poisons, and also indirectly via single-stranded DNA (ssDNA) lesions which induce replication forks collapse, leading to DSB formation (13).

## Homologous Recombination and Chemotherapeutic Compounds Targets

**Figure 1:**
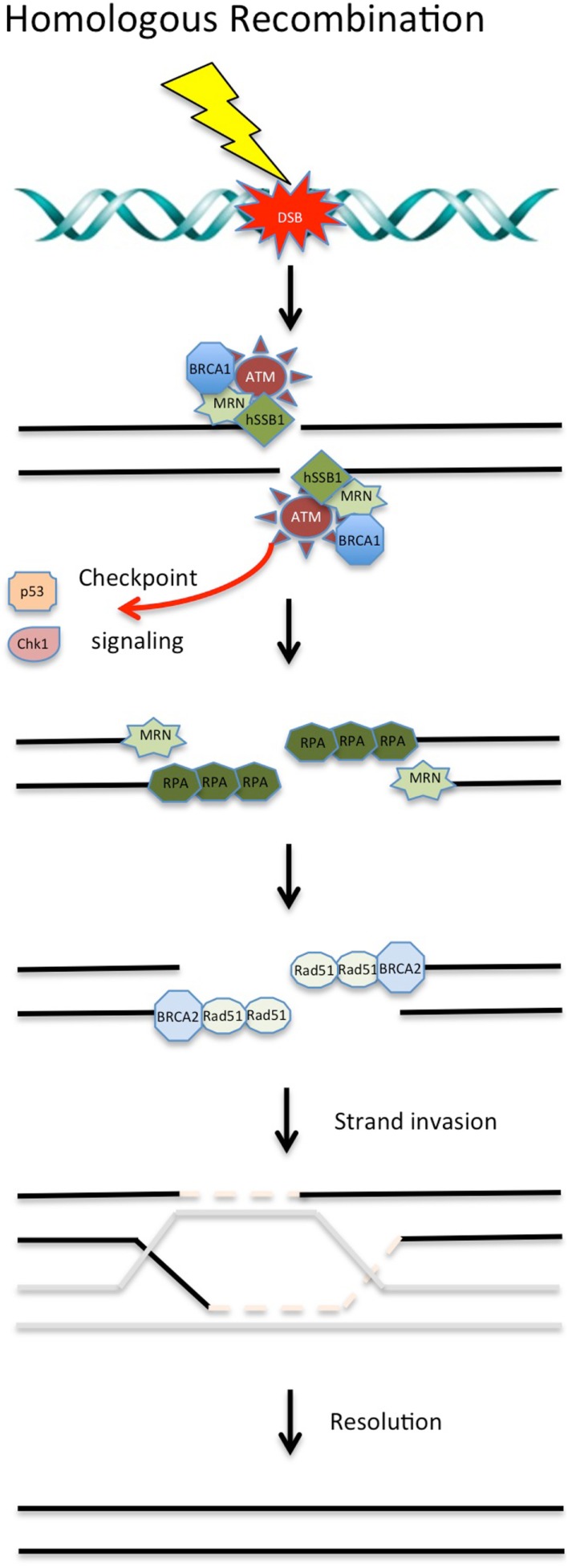
**DNA double-strand break repair via homologous recombination**. During S and G2 phases of the cell cycle, DSBs can be repaired via HR using a sister chromatid. Targeting of these proteins involved in HR with chemotherapeutic compounds shows promise in the clinical setting. See text for details.

Following the induction of a DSB, the Mre11/Rad50/NBS1 (MRN) complex is recruited to the break site by human single-stranded binding protein 1 (hSSB1) (14–16). MRN binds to the DNA surrounding the lesion and resects the DNA around the break in a 5′–3′ dependent direction. This acts as a signal to recruit other DDR proteins. This resection by the MRN complex is stimulated in the early stages of HR by an interaction with CtIP (17, 18). Following initiation of resection by Mre11, Exo1 performs more extensive resection to expose long stretches of ssDNA (19–21). Replication protein A (RPA), a ssDNA binding heteromeric complex, binds to the exposed ssDNA and is retained at the lesion site by BRCA1 (22). The binding of RPA to the ssDNA substrate ensures that secondary structures are not formed in the DNA and protects the ssDNA from nucleases. RPA is displaced from the DNA by the recombinase Rad51, which is loaded by BRCA2. Rad51 forms a nucleoprotein filament along the ssDNA and functions to allow strand invasion of the sister chromatid (23). Once the DSB is resolved, the DNA is ligated together, completing the process of HR (24). There are a number of key proteins involved in HR that are currently therapeutic targets or have been identified as potential targets (see Figure 1).

### The MRN complex

Human single-stranded binding protein 1 serves as the primary sensor of DSBs and is also involved in the early steps of HR through the recruitment of the MRN complex (14–16). Once recruited, the MRN complex specifically functions in the resection of DNA ends, activates the ATM kinase, and subsequently activates the cell cycle checkpoints (25). The human syndromes that result from mutations in each component in the MRN complex highlight the requirement of the MRN complex for the maintenance of genomic stability: NBS (26), AT-like disorder (AT-LD) (27), and NBS-like NBS disorder (28) result from NBS1, Mre11, and Rad50 mutations, respectively. The MRN complex is also indispensible in development, as null-mutations of these genes cause embryonic lethality in mice (29–31).

A study using a forward genetic screen identified a specific small molecule inhibitor of the MRN complex, dubbed Mirin. Mirin inhibits MRN-dependent ATM activation and disrupts the endonuclease activity of Mre11, which leads to the failure of the G2/M checkpoint and HR repair (32). More recently, Mirin was also shown to effect DSB repair via NHEJ (33). Despite the promise shown in early studies, at the time of writing this review, Mirin has not progressed to assessment in the clinic.

The use of retroviral gene therapy using recombinant adenovirus with mutant forms of the individual proteins in the MRN complex has shown promising results *in vitro* and *in vivo*. In human head and neck squamous cell carcinoma cell lines, expression of adenoviral mutant NBS1 significantly increases cisplatin-induced DSBs and cytotoxicity. This suggests that tumor chemosensitization occurred through the increase of DSBs, as the MRN complex was not able to detect the breaks (34). A novel dominant-negative adenoviral vector containing a mutant Rad50 gene significantly down regulated MRN expression and markedly disrupted MRN function in human squamous cell carcinoma cells. A combination of cisplatin and mutant Rad50 gene therapy produced significant tumor cytotoxicity *in vitro*, with a corresponding increase in DNA damage and telomere shortening. In cisplatin-resistant human squamous cell cancer xenografts, this combination therapy caused dramatic tumor regression with increased apoptosis (35). Further studies have shown this method is effective *in vivo*, however clinical trials using this method of radiosensitizing have not progressed at present.

Telomelysin is a type 5-adenovirus in which the genes have been modified to be able to selectively replicate in cancer cells. The replication of telomelysin is controlled by the human telomerase reverse transcriptase promoter and has been shown to be effective in sensitizing cells to IR (36). The radiosensitivity was due to inhibition of the MRN complex *in vivo* (37). It was found that the expression of the adenoviral E1B55 kDa protein lead to the degradation of the MRN complex (38). A Phase I clinical trial studying telomelysin demonstrated it was effective in various solid tumors and was well tolerated without any adverse effects to patients (39). A Phase I/II trial for the effects of telomelysin on esophageal cancer has commenced in Japan and a Phase I/II clinical trial on liver cancer is planned in the near future.

Resveratrol is a naturally occurring polyphenol that is present in more than 72 plant species. Resveratrol has been shown to arrest the cell cycle (40), promote cellular differentiation (41), and induce apoptosis (42). However, the precise mechanism for these effects remains to be elucidated. A recent gene expression analysis of breast cancer cells treated with resveratrol identified decreased expression of Mre11 and NBS1, key components of the MRN complex. A number of other proteins involved in HR were also down regulated, including BRCA2 and Rad51, whereas Rad52 was up-regulated (43). This suggests resveratrol may function through a number of mechanisms including the MRN complex. *In vivo* studies showing positive, neutral, as well as negative outcomes depending on dose, administration method, and cancer type (44). There have been 76 clinical trials using resveratrol listed at clinicaltrials.gov. Further studies need to be performed to determine if resveratrol can be used for human cancer prevention or therapy and also determine the exact mechanism of the radiosensitizing properties of resveratrol.

### Ataxia telangiectasia mutated

As discussed above, the MRN complex is responsible for the activation of ATM, a major kinase in the DDR. ATM is a member of the phosphoinositide 3-kinase-related kinase (PIKK) family, which also includes ataxia telangiectasia and Rad3-related (ATR) and DNA-dependent protein kinase catalytic subunit (DNA-PKcs). The MRN complex activates ATM in response to DSBs by recruiting it to the sites of damage (45). Activated ATM is responsible for the induction of the G1/S, intra-S, and G2/M checkpoints, via the phosphorylation of a number of down-stream effector kinases and transcription factors, including p53 and p21 (46). The activation of the cell cycle checkpoints is critical in the DDR to allow for DNA repair to occur before the cell divides ensuring genomic stability.

A study showed that ATM is responsible for hundreds of phosphorylation events in the cell in response to DNA damage, highlighting the key role this kinase plays in the DDR (47). ATM is also required for the full activation of Akt (also known as protein kinase B) in response to insulin in the cytoplasm (48). ATM has also shown to be involved in the regulation of the expression and stability of ribonucleotide reductase and the mitochondrial homeostasis through the control of mitochondrial DNA (mtDNA) copy number dynamics and expression (49). This link with ATM and the regulation of mtDNA may be involved in the resistance of genotoxic stress, highlighted by the potential role of the nuclear co-activators peroxisome proliferator-activated receptor gamma co-activator-1β in DNA damage repair (50). These key roles in the DDR have ensured that ATM has been a prime candidate for inhibition in cancer treatment and further investigations into synthetic lethality for AT patients may show promise.

Caffeine and wortmannin were the first ATM inhibitors identified in the lab and were shown to increase sensitivity to radiation and chemotherapeutic compounds (51, 52). Both caffeine and wortmannin were later shown to be non-specific inhibitors of ATM and also inhibited the other PIKK, ATR, and DNA-PKcs and the potency of these drugs rendered them unsuitable for use in a clinical setting.

The flavonoid quercetin was identified as an inhibitor of phosphoinositide 3-kinase (PI3K) with an IC_50_ of 3.8 μM (53). Analogs of quercetin were synthesized and investigated for their inhibition of PI3K, which led to the discovery of LY294002, an ATP-competitive inhibitor (54). LY294002 was later found to also inhibit ATM (55) and DNA-PKcs (56). However, in the high doses required to inhibit these proteins (>10 μM), LY294002 targeted several unrelated proteins including calcium channels and the estrogen receptor (57, 58).

Despite its lack of specificity, LY294002 was used as a research tool to identify more specific PIKK inhibitors. A study screening a drug-library based on LY294002 identified KU-55933, a small molecule ATP-competitive inhibitor, which was specific to ATM (55). KU-55933 is a potent inhibitor of ATM with an IC_50_ of 0.013 μM and is highly specific to ATM compared to other PI3K and PIKK’s. This compound effectively sensitized tumor cells to radiation and DSBs inducing chemotherapeutic compounds, such as camptothecin and etoposide, and initial work has shown this compound may be used as a potential clinical treatment (55).

KU-60019, an improved analog of KU-55933 has been shown to inhibit ATM with an IC_50_ of 0.0063 μM and also inhibits migration and effectively radiosensitizes human glioma cells (59). Further studies on KU-60019 are currently being performed, specifically as a radiosensitizer with standard chemotherapy regimes on glioblastoma in preparation for clinical trials (60).

Another ATM specific inhibitor, CP466722, was identified in a targeted compound library screen, looking for inhibitors of ATM-dependent phosphorylation events *in vitro*. *In vivo* treatment with CP466722 resulted in transient inhibition of ATM and sensitized cells to IR however, upon removal, ATM kinase activity and the subsequent phosphorylation of down-stream targets was completely restored (61). The clinical implications of this transient inhibition of ATM, requires further study.

KU59403 is the latest of the ATM inhibitors that have been studied and one that shows the most promise for clinical trials in patients. KU59403 increased the cytotoxicity of the topoisomerase I and II poisons camptothecin, etoposide, and doxorubicin, *in vitro*, in a non-p53-dependent manner. Importantly, upon injection, KU59403 was seen to be distributed to tissues in mice at concentrations required to inhibit ATM activity, these were shown to be maintained for at least 4 h in colon cancer tumor xenografts and enhanced the anti-tumor activity of topoisomerase poisons. This chemosensitization was both dose and schedule-dependent and provided the first proof-of-principle pre-clinical data to support future clinical development of ATM inhibitors (62). However, at present, there are no reports of ATM inhibitors in use in clinical trials.

It should be noted that CGK-733, a small molecule that was initially reported to inhibit both ATM and ATR kinase activities and block checkpoint signaling with great selectivity, was later retracted (63). Further studies were completed showing that CGK-733 has no specific inhibitory effect on ATM or ATR (64). Unfortunately, the compound is still being marketed as an ATM/ATR inhibitor.

### hSSB1/2

Human single-stranded binding protein 1 is required for the activation of ATM through the recruitment of the MRN complex to the break site (14–16). Until the identification of hSSB1 and hSSB2, as a DNA single strand-binding proteins, RPA was the only known eukaryote member of the single strand-binding protein family (SSB) to be involved in DNA repair (14). As mentioned above, hSSB1 is required for the efficient recruitment of the MRN complex to sites of DSBs and for the efficient initiation of ATM-dependent signaling (15, 16). hSSB1 binds directly to the MRN complex through NBS1 and functions to also stimulate its nuclease activity. Identification of specific chemotherapeutic compounds to target hSSB1 and hSSB2 will enable the sensitization of cancer cells to radiotherapy and these are currently being explored by our laboratory and by industry.

### Chk1/2

The checkpoint kinases, Chk1 and Chk2, are critical for cell cycle activation following the induction of DSBs and serve to maintain the genomic integrity of cells (65). This cell cycle checkpoint activation is achieved through maintaining or augmenting the inhibitory phosphorylation of the cyclin-dependent kinases (CDKs) by inhibiting the CDC25 phosphatases. Specifically, phosphorylation of CDC25A is required for the initiation of the S-phase checkpoint and phosphorylation of CDC25C for the G2/M checkpoint (66). Both Chk1 and Chk2 are also required for the activation or inhibition of a number of other cell cycle checkpoint proteins and tumor suppressor proteins, including p53 (67).

Of these checkpoint kinases, Chk1 is critical for the induction of HR, as inhibition of Chk1 *in vitro* lead to persistent unrepaired DSBs and cell death (68). Rad51 was also shown to be recruited to DSBs at replication forks in a Chk1-dependent manner, therefore Chk1 is essential for HR at stalled replication forks. Highlighting this critical role, Chk1 null mice were found to be embryonically lethal (69). Due to its critical role in HR, this review will focus on Chk1 inhibitors only, however a number of these Chk1 inhibitors are also known to target Chk2.

UCN-01, a staurosporine inhibitor was the first inhibitor identified for both Chk1 and Chk2 and treatment with this compound lead to G2/M checkpoint deficiencies in IR-treated p53-deficient tumor cells (70). However, due to the broad spectrum of targets for UCN-01, including protein kinase C, CDK, and CDK2, the use of UCN-01 presented challenges in the clinical setting (71). Since the discovery of UCN-01, increasingly specific inhibitors for Chk1 have been identified.

XL-844, also known as EXEL-9844, is a potent, ATP-competitive inhibitor of Chk1 and Chk2. *In vitro*, XL-844 showed limited ability as a monotherapy but substantially enhanced gemcitabine-induced cell killing. XL-844 increased the gemcitabine-induced DNA damage, blocked CDC25A phosphorylation, abrogated the gemcitabine-induced S-phase checkpoint, and induced premature mitotic entry. Interestingly, XL-844 also induced phosphorylation of Chk1 (72). However, a further *in vitro* study showed that, in response to IR, Chk2, rather than Chk1 appeared to be activated by irradiation and this activation was suppressed by XL-844 (73). A Phase I clinical trial of XL-844 as a monotherapy or in conjunction with gemcitabine in patients with advanced malignancies was discontinued before completion (clinicaltrials.gov – NCT00475917).

AZD7762 is a potent ATP-competitive checkpoint kinase inhibitor that was identified via a compound library screen using Chk1. AZD7762 was found to inhibit both Chk1 and Chk2 with *in vitro* and *in vivo* studies confirming the abrogation of the checkpoint response to gemcitabine (74). *In vivo* studies involving human breast cancer xenografts in mice demonstrated that AZD7762, in combination with irinotecan, improved host survival and reduced tumor growth selectively in p53 mutant tumors (75). A Phase I clinical trial to evaluate the safety, tolerability, and pharmacokinetics of AZD7762, as a monotherapy or in conjunction with gemcitabine, in patients with advanced solid malignancies has just been completed, however no published data have been released as yet (clinicaltrials.gov – NCT00413686). However, interestingly two Phase I trial of AZD7762 in conjunction with irinotecan (clinicaltrials.gov – NCT00473616) and gemcitabine (clinicaltrials.gov – NCT00937664) were terminated early. Trial NCT00937664 was terminated due to incidence of cardiac toxicities reported in the overall Phase I development program (76). This result may affect further clinical development of AZD7762.

PF-00477736 is a potent, selective ATP-competitive small molecule inhibitor of Chk1 and was shown to abrogate cell cycle arrest induced by DNA damage and enhanced the cytotoxicity of clinically important chemotherapeutic agents, including gemcitabine and carboplatin both *in vitro* and *in vivo* in mouse xenografts (77). In combination with docetaxel, PF-00477736 was found to abrogate the DNA damage checkpoints and resulted in sensitization to docetaxel (78). The only clinical trial to date with PF-00477736 looked at the effects in combination with gemcitabine in advanced solid tumors, however this study was prematurely terminated (clinicaltrials.gov – NCT00437203).

SCH900776, also known as MK-8776, was identified as a highly potent Chk1 inhibitor using a high-content, cell-based screen for γ-H2AX induction (γ-H2AX is a surrogate marker for double-strand DNA breaks). SCH900776 also enhanced the anti-tumor effects of gemcitabine *in vivo* (79). SCH900776 was also shown to inhibit CDC25C degradation, abrogates S-phase arrest, and induces DNA damage (80). When SCH900776 was combined with low concentrations of hydroxyurea, both p53-deficient and p53-proficient cell lines were sensitive to the combination (81). It was also demonstrated, *in vitro*, that some cell lines were highly sensitive to SCH900776 alone. *In vivo* models with a human pancreas tumor xenografts mouse model combined SCH900776 with gemcitabine, this showed a significantly delayed tumor growth compared to either drug alone (82). A Phase I clinical trial was undertaken using SCH900776 in combination with cytarabine in patients with acute leukemia. This trial indicated that SCH900776 was tolerated by patients and progressed to a Phase II clinical trial (83). A randomized Phase II clinical trial is currently recruiting to study how well cytarabine, with or without SCH900776, works in treating adult patients with relapsed acute myeloid leukemia (clinicaltrials.gov – NCT01870596).

LY2603618, a pyrazinyl-urea compound, was identified as a Chk1 inhibitor via *in vitro* kinase assays. LY2603618 is currently being investigated in a number of Phase I and II clinical trials and pre-clinical data on their effects *in vitro* and *in vivo* have been recently released. LY2603618 was shown *in vitro* to produce a cellular phenotype similar to that reported for depletion of Chk1 by siRNA and impaired DNA synthesis, elevated H2AX phosphorylation, which is indicative of DNA damage, and caused premature entry into mitosis. *In vivo* treatment of human mutant p53 lung cancer cell xenografts in mice, with gemcitabine, resulted in a stimulation of Chk1 kinase activity that was inhibited by co-administration of LY2603618 (84). In a Phase 1 dose escalation clinical trial of LY2603618 combined with pemetrexed, 9 out of 31 patients achieved stable disease and 1 pancreatic cancer patient had a partial response (85). Two other clinical trials have been completed using LY2603618, in conjunction with gemcitabine, in patients with pancreatic cancer (clinicaltrials.gov – NCT00839332); and an open-label study in patients with advanced and/or metastatic solid tumors (clinicaltrials.gov – NCT01296568), however no results have been published on these clinical trials. There are currently two active clinical trials currently underway studying LY2603618: the first is studying the safety and tolerability of LY2603618 in combination with gemcitabine in patients with solid advanced or metastatic tumors (clinicaltrials.gov – NCT01341457) and the second is investigating the safe dose of LY2603618 that can be combined with pemetrexed and cisplatin and to test if this triplet offers a significant improvement in progression-free survival in participants with Stage IV non-squamous non-small cell lung cancer (clinicaltrials.gov – NCT01139775).

LY2606368 has been identified as a Chk1 inhibitor by Eli Lilly and a Phase I clinical trial is currently recruiting to investigate the effects of LY2606368 in patients with advanced solid tumors (clinicaltrials.gov – NCT01115790), however no pre-clinical data are available for this inhibitor.

Two recently identified Chk1 inhibitors, GDC-0425 and GDC-0575, are currently undergoing clinical trials, however again no pre-clinical data are available. Both compounds are currently being evaluated for the safety, tolerability, and pharmacokinetics administered as a monotherapy or in combination with gemcitabine in patients with refractory solid tumors or lymphoma: GDC-0425 (clinicaltrials.gov – NCT01359696); and GDC-0575 (clinicaltrials.gov – NCT01564251).

SAR-020106 is a novel, selective, and potent ATP-competitive inhibitor of Chk1. SAR-020106 has been shown to abrogate the etoposide-induced G2 arrest and significantly enhances the cell killing of gemcitabine *in vitro* and in a p53-dependent fashion. *In vivo*, it was found that irinotecan, gemcitabine, and radiation activity was enhanced by SAR-020106 with minimal toxicity (86, 87). Whilst SAR-020106 has not undergone any clinical trials at present, this pre-clinical data suggest it is a prime candidate for investigation in p53-defective tumors.

### p53

The tumor suppressor protein p53 is a transcriptional regulator of a number of genes involved in DNA repair, cell cycle progression, and apoptosis. Highlighting this function, p53 was found to be mutated in approximately 50% of cancers (88). p53 is activated in response to DNA damage by phosphorylation by the ATM, ATR, Chk2, and Chk1 kinases and these phosphorylation events allow for the stabilization of p53 and activate its transcriptional functions allowing the regulation of a number of genes responsible for cell cycle progression and apoptosis (89).

Initial work on p53-mutated cancers has investigated restoring the function of p53, thus leading to effective apoptosis in response to the chemotherapy. Two main mechanisms have been investigated – restoring p53 to cancer cells using a recombinant adenovirus encoding p53 or using small compounds or short peptides to restore the activity of p53.

Pre-clinical *in vitro* and *in vivo* studies of adenovirus-mediated p53 (Ad-p53) cancer gene therapy showed promising results with advexin (90), gendicine (91), and SCH-58500 (92). Initial clinical trials with these Ad-p53 vectors showed that administration was a safe, feasible, and effective strategy against many types of cancers, however, the anti-tumor efficacy has been limited in some cancer patients. These Ad-p53 vectors have also been used in combination with conventional DNA-damaging treatments, indicating the induction of the apoptotic pathway via Ad-p53 can restore the sensitivity to radiation and chemotherapy in some resistant tumors.

However, issues exist with the low transduction of p53 into cancer cells via these Ad-p53 vectors, to overcome this replication, competent oncolytic adenoviruses have been developed. The CRAd-p53 vector has been used where the promoters of cancer-related genes are used to regulate virus expression in a tumor-dependent manner. Recent work has focused on AdDelta24-p53 (93), SG600-p53 (94), and OBP-700 (95). Initial *in vitro* and *in vivo* studies have shown these CRAd-p53 vectors are a safe and effective therapy for inducing anti-tumor effects and have been shown to induce higher p53 expression and stronger anti-tumor effects than the Ad-53 vectors, highlighting their potential in future clinical trials.

A number of small compounds and peptides have been shown to be effective in restoring the function of p53 in tumor cells, including CP-31398 (96), PRIMA-1 (97), CDB3 (98), peptide 46 (99), and SCH529074 (100). These small compounds and peptides act to stabilize p53 in its active biological conformation, thus restoring its transcriptional activity. Initial *in vitro* work on these small compounds and peptides have shown promising results and further *in vivo* studies are required to determine their efficacy before clinical trials can commence.

### Replication protein A

Due to its key role in DNA replication and repair, via HR and NER, RPA has been the subject of a number of studies to identify potential inhibitors. RPA is over-expressed in a number of cancers, including colon (101), esophageal (102), and breast (103). RPA is a heterotrimeric protein, consisting of RPA1 (p70), RPA2 (p32), and RPA3 (p14) subunits. RPA protects ssDNA from nucleolytic attack, prevents DNA hairpin formation, and blocks DNA reannealing by binding directly to the ssDNA through four OB-folds. After DNA damage, RPA coats ssDNA and enhances the capacity of Rad51 oligomer formation at sites of damage (104).

Initial work has investigated the disruption of the DNA binding capacity of RPA and also inhibition of its protein partner interactions using small molecule inhibitors. Selective inhibition of both the protein binding and DNA binding capacity of RPA has the potential to inhibit the DDR and to sensitize cancer cells to DNA-damaging agents.

TDRL-505, a novel small molecule inhibitor, has recently been shown to inhibit the RPA–DNA interaction, thereby preventing cell cycle progression, induces cytotoxicity, and increases the efficacy of the chemotherapeutic DNA-damaging agent, cisplatin, *in vitro* (105). TDRL-505 inhibits the DNA binding capacity of RPA by blocking the OB-folds of RPA1. Further studies need to be completed in mouse models to determine the efficacy of this compound.

Isobornyl derivatives have also been shown to be RPA inhibitors in a screen of the National Cancer Institute library, with CheSS19 shown to interact irreversibly with the OB-folds of RPA1 (106). MCI13E, a haloester modified form of CheSS19, decreased cell viability and induced apoptosis, showing synergistic effects with cisplatin in lung cancer cells (107). However, this compound did not affect the DNA binding capacity of RPA, but instead may act through the alkylation of cysteine residues of RPA. Further studies, both *in vitro* and *in vivo* are required to fully understand the mechanisms of RPA inhibition by MCI13E prior to clinical studies being undertaken.

The initiation of the DDR by RPA is also mediated by protein–protein interactions involving the N-terminal domain of the p70 subunit with partner proteins, including the MRN complex (108), Rad51 (109), and BRCA2 (110). Inhibition of these interactions increases sensitivity toward DNA damage and replication stress and may therefore be a potential strategy for cancer drug discovery. Combining RPA inhibition with radiation therapy could lead to increased cytotoxicity in tumor cells via inhibition of DNA DSB repair via NHEJ or HR, both of which have been shown to require RPA.

### Rad51

Rad51 plays an important role in maintaining genome stability through the HR pathway in response to DNA damage. This is highlighted by the fact that Rad51 knockout mice show early embryonic lethality (111). Rad51 is a DNA recombinase and polymerizes onto resected DNA ends to form a nucleoprotein filament that promote strand invasion and exchange between homologous DNA duplexes (112). It is suggested the improper regulation of Rad51 may affect tumorigenesis, as Rad51 has been shown to be over-expressed in a number of cancer phenotypes, including esophageal, pancreatic, lung, leukemia, and head and neck cancers. Conversely, Rad51 is also under-expressed in a number of cancer cells. This variable expression of Rad51 has been shown to promote the resistance of tumors to chemotherapy (113). Using antisense RNA or RNAi to deplete the levels of Rad51 *in vitro* has been shown to sensitize tumor cells to chemotherapy agents, including cisplatin (114). These effects of Rad51 depletion demonstrate the potential of Rad51 inhibitors in cancer therapy.

The first identification of a Rad51 inhibitor was a small peptide, homologous to the BRC-motif of the BRCA2 protein, which was found to bind Rad51, thereby preventing its DNA binding capacity (115). This peptide inhibited the formation of Rad51 nuclear foci and disrupted HR *in vitro*. Using an *in silico* approach on the BRC domains of BRCA2, a chimeric peptide with an efficiency 10 times higher than the original peptide was identified (116). This new peptide inhibited Rad51 DNA binding and DNA strand exchange activity however, although these peptides are currently being used as a research tool they have not yet found clinical applicability. Further investigation of peptides and peptidomimics inhibiting Rad51 function may elucidate novel inhibitors targeting the HR pathway in tumors. However, this approach still holds drawbacks mainly due to the pharmacokinetics of peptide-based inhibitors and administration of these agents may not be optimal in a clinical setting.

More recently, a DNA strand exchange assay was performed and used to identify Rad51 inhibitors by high-throughput screening of the NIH small molecule repository. This study identified 17 potential inhibitors, of which 3 were studied further. Compound B02 was identified that specifically inhibited human Rad51 with two other compounds, A03 and A10, which inhibited both Rad51 and RecA, but not the structurally unrelated Rad54 protein. B02 directly interacts with Rad51 and disrupts its binding to DNA and nucleoprotein filament formation. The interaction of B02 with Rad51 disrupted DSB-induced HR and enhanced the sensitivity of cells to cisplatin (117). Further work on these compounds, both *in vitro* and *in vivo*, are required before they can be introduced into clinical trials.

A small molecule inhibitor to Rad51 was recently identified through a high-throughput screen of a library of 10,000 small molecules (118). The molecule RI-1 covalently binds to Rad51, thereby inhibiting its ability to form filaments on ssDNA. RI-1 inhibits the nuclear foci of Rad51 at sites of DNA damage and sensitizes various cancer cell types to cross-linking chemotherapy, but did not affect Rad51 protein levels. There are limits to the development of RI-1 in pre-clinical *in vivo* models due to its short half-life in tissue culture media and aqueous buffers. RI-2, a homolog of RI-1, was created that mitigated these effects (119). RI-2 was shown to bind Rad51 and inhibit the nuclear foci of Rad51 at sites of DNA damage. RI-2 is currently the subject of further *in vitro* and *in vivo* studies and is being used to identify third generation analogs that inhibit the function of Rad51.

A further screen using a yeast-2 hybrid system identified a phenylsulfonyl indolyl isoquinoline compound, IBR2, as a Rad51 inhibitor. IBR2 functions to block Rad51 multimerization, accelerating proteasome-mediated Rad51 protein degradation, and thus impairing IR-induced Rad51 foci formation in the nucleus and HR activity. IBR2 inhibited cancer cell growth and induced apoptosis (120). A synergistic cell-killing effect was produced with a combination of IBR2 and imatinib *in vitro*. *In vivo* studies involving breast cancer xenografts in nude mice showed significantly inhibited tumor growth with no apparent secondary physiological abnormalities. Further studies on the effects of IBR2 are required before moving into a clinical trial.

### BRCA1/2

The breast cancer susceptibility proteins, BRCA1 and BRCA2, have a key role in efficient HR response to DSBs. Mutations in these genes greatly increase the susceptibility to cancer, especially breast, ovarian, and prostate. Mutations in the BRCA genes are responsible for the increased risk of breast cancer, specifically 59–87 and 38–80% for BRCA1 and BRCA2 mutations respectively. BRCA1 functions in both checkpoint activation and also in the early steps of HR, by controlling DNA resection (121). BRCA2 functions in Rad51 transport and loading (122). Both BRCA1 and BRCA2 are required for normal embryonic development in mice (123, 124).

Direct inhibition of BRCA1 and BRCA2 in tumors is generally problematic due to the wide expression of these proteins in most tissues and inhibition may lead to other issues, including cancer development in healthy tissue. One approach is the possible up regulation of the BRCA1 and BRCA2 proteins, however there is no data to suggest that up regulation blocks tumorigenesis.

### Poly ADP-ribose polymerase 1

Most of the work in the BRCA therapeutic research area has focused on tumors that are known to have mutations in BRCA1 or BRCA2. Using synthetic lethality, these studies have focused on disrupting complimentary pathways to repair DNA damage, with the most interesting results coming from poly ADP-ribose polymerase 1 (PARP1) inhibitors. PARP1 is involved in DNA repair, replication, transcriptional regulation, chromatin modification, and apoptosis (125, 126). In regards to DNA repair, PARP1 is involved in BER which repairs DNA damage due to reactive oxygen species and alkylation (127). Inhibition of this pathway, taken together with a loss of HR due to BRCA mutations, creates a synthetic lethality, which can be exacerbated when used in conjunction with chemotherapy agents.

However, PARP1 inhibition and the subsequent synthetic lethality can be used on other cancers that do not have mutations in BRCA1 or BRCA2 but that have a defect in the HR pathway, including mutations in ATM, Chk2, Rad51, and NBS1. However, NHEJ could compensate for the loss of HR in these cells. Further studies have identified that PARP1 normally functions to promote HR by suppressing various components of the NHEJ pathway (128). Inhibition of PARP1 would therefore lead to increased NHEJ, a more error-prone repair mechanism than HR, this would increase chromosome instability in response to chemotherapy and during S-phase of the cell cycle at stalled DNA replication forks.

Poly ADP-ribose polymerase catalyzes the cleavage of NAD+ to nuclear acceptor proteins, leading to the formation of ADP-ribose polymers, realizing nicotinamide in the process. Nicotinamide was the first PARP inhibitor identified, although it was not potent (129). Analogs of nicotinamide, including 3-aminobenzamide, were the first generation of PARP inhibitors (130).

The first clinical trial targeting PARP1 in BRCA populations was with the oral drug olaparib, also known as AZD2281, as a monotherapy (131). Olaparib achieved encouraging response rates of 41 and 33% in patients with BRCA1 or BRCA2 mutations, respectively. Olaparib was also used in a combined therapy with carboplatin *in vivo* and showed a profound decrease in tumor growth and increase in patient survival (132). A Phase II trial with olaparib was conducted on patients with advanced BRCA1/2 mutant breast cancers (133) and ovarian cancers (134). Both of these studies showed a dose-dependent effect of olaparib. Currently, there is a Phase III olaparib trial being undertaken by AstraZeneca. This trial aims to determine the benefit, by progression-free survival, of olaparib as a maintenance monotherapy, in BRCA mutated ovarian cancer patients, who are in complete or partial response following platinum-based chemotherapy.

Another PARP inhibitor is veliparib. An *in vivo* study of veliparib, also known as ABT-888, confirmed the PARP inhibitory effects in paired tumor biopsies and peripheral mononuclear cells (135). A number of Phase I trials have been conducted with veliparib in combination, including topotecan (136) amongst others. Many of these trials have shown promising results, however myelosuppression, where bone marrow activity is decreased, has been shown as the most common adverse event observed.

Iniparib, also known as BSI-201, was the first PARP inhibitor to undergo Phase III clinical trials after showing promising results in randomized Phase II trials in patients with triple-negative breast cancer (137). The results of the subsequent Phase III clinical trial were not as expected, missing the co-primary endpoints of overall survival and progression-free survival. However, very little pre-clinical data on the effects of iniparib were published before clinical trials began and iniparib was shown not be related to other PARP inhibition and showed very low PARP inhibition *in vitro* (138, 139).

Rucaparib, also known as AG014699, was used in a Phase I clinical trial in combination with temozolomide (140). Rucaparib was well tolerated in patients and showed promising results for assumed HR-deficient tumors (based on tumor type). Rucaparib was also used as a monotherapy in a Phase I/II clinical trial with patients with solid tumors. Rucaparib was well tolerated in this trial and showed promising results (clinicaltrials.gov – NCT01482715). A subsequent Phase II clinical trial, with patients with melanoma, was conducted in combination with temozolomide, and showed an objective response rate with 17.4 and 36% of patients remaining progression-free after 6 months (141).

A therapeutic index-based strategy was used to identify CEP-8983, a novel 4-methoxy-carbazole inhibitor of PARP1 and PARP2 (enzyme IC_50_ values of 20 and 6 nmol/L, respectively). CEP-8983 was found to cause significant sensitization of chemotherapy-resistant tumor cell lines to the effects of temozolomide and camptothecin *in vitro*. Administration of CEP-8983, delivered orally in the form of CEP-9722, attenuated *in vivo* PARP activity and resulted in significant chemosensitization of temozolomide and irinotecan in chemotherapy-resistant tumor xenografts (142). A Phase I clinical trial with CEP-9722, used as a monotherapy or in conjunction with temozolomide, was recently completed with patients with advanced solid tumors (clinicaltrials.gov – NCT00920595). A Phase I/II clinical trial using CEP-9722 on solid tumors is currently underway (clinicaltrials.gov – NCT01311713).

MK-4827, also known as niraparib, is a novel 2-phenyl-2H-indazole-7-carboxamide PARP inhibitor that displayed anti-proliferation activities against BRCA1- and BRCA2-deficient cancer cells *in vitro*. MK-4827 was found to be well tolerated *in vivo* and demonstrated efficacy as a single agent in a xenograft model of BRCA1-deficient cancer (143). A Phase I clinical trial of patients with solid tumors using MK-4827 was shown to have favorable pharmacokinetics, inhibited PARP activity effectively, is well tolerated and has anti-tumor activity in carriers of BRCA1 and BRCA2 mutations and patients with sporadic cancers (144).

BMN 673, an inhibitor of PARP catalytic activity, has exhibited selective anti-tumor activity at much lower concentrations (IC_50_ = 0.57 nM) than the earlier generation of PARP inhibitors, including olaparib, rucaparib, and veliparib. BMN 673 is readily orally bioavailable and *in vivo* studies with xenograft tumors carry defects in BRCA1/2 or PTEN were sensitive to BMN 673. Synergistic effects were observed when BMN 673 was combined with temozolomide, SN38, or platinum drugs (145). A number of pre-clinical studies and Phase 1, Phase II, and Phase III clinical trials utilizing BMN 673 as a monotherapy or in conjunction with various drugs, are currently underway.

It is important to note that not all breast cancer patients with BRCA mutations responded to PARP inhibition (131) and a substantial number of patients with advanced BRCA1-mutant cancers are resistant to these agents. Further studies on PARP inhibitors, along with the current clinical trials, are needed to assess the efficacy of PARP inhibition in BRCA mutant and other HR-defective cancers in conjunction with chemotherapy or as a monotherapy.

A list of all chemotherapeutic compounds targeting the HR pathway is provided in Table 1.

**Table 1:** **Chemotherapeutic compounds targeting the homologous recombination DNA repair pathway**.

Compound	Class	Clinical phase	Combination
Mirin	MRN complex	–	–
Adenoviral mutant NBS1	NBS1	–	Cisplatin
Adenoviral mutant Rad50	Rad50	–	Cisplatin
Telomelysin	MRN complex	I/II	Monotherapy
Resveratrol	MRN complex	–	–
Caffeine	PIKK	–	–
Wortmannin	PIKK	–	–
Quercetin	PI3K	–	–
LY294002	ATM and DNA-PKcs	–	–
KU-55933	ATM	–	–
KU-60019	ATM	–	–
CP466722	ATM	–	–
KU59403	ATM	–	–
UCN-01	Chk1 and Chk2	I	Monotherapy or topotecan or cisplatin
XL-844	Chk1 and Chk2	I	Monotherapy or gemcitabine
AZD7762	Chk1 and Chk2	I	Monotherapy or gemcitabine or irinotecan
PF-00477736	Chk1	I	Gemcitabine
SCH900776	Chk1	I/II	Monotherapy or cytarabine or gemcitabine
LY2603618	Chk1	I/II	Gemcitabine or pemetrexed and cisplatin
LY2606368	Chk1	I	Monotherapy
GDC-0425	Chk1	I	Monotherapy or gemcitabine
GDC-0575	Chk1	I	Monotherapy or gemcitabine
SAR-020106	Chk1	–	–
Advexin	p53	I/II	Monotherapy and chemotherapy drugs
SCH-58500	p53	I/II	Monotherapy and chemotherapy drugs
AdDelta24-p53	p53	–	–
SG600-p53	p53	–	–
OBP-700	p53	–	–
CP-31398	Stabilizes p53	–	–
PRIMA-1	Stabilizes p53	–	–
CDB3	Stabilizes p53	–	–
Peptide 46	Stabilizes p53	–	–
SCH529074	Stabilizes p53	–	–
TDRL-505	RPA	–	–
CheSS19	RPA	–	–
MCI13E	RPA	–	–
B02	Rad51	–	–
A03	Rad51 and RecA	–	–
AI-10	Rad51 and RecA	–	–
RI-1	Rad51	–	–
RI-2	Rad51	–	–
IBR2	Rad51	–	–
3-Aminobenzamide	PARP1	–	–
Olaparib	PARP1	I/II/III	Monotherapy
Veliparib	PARP1	I	Topotecan or carboplatin or doxorubicin or irinotecan
Rucaparib	PARP1	I/II	Monotherapy or temozolomide
CEP-9722	PARP1 and PARP2	I/II	Monotherapy or temozolomide
MK-4827	PARP1	I	Monotherapy or temozolomide
BMN 673	PARP1 and PARP2	I/II/III	Monotherapy or temozolomide or irinotecan

## Non-Homologous End Joining and Chemotherapeutic Compounds Targets

### Classical-NHEJ

The classical-NHEJ (C-NHEJ) pathway is the major pathway of DSB repair [reviewed in Ref. (146)], estimated to rapidly repair up to 85% of IR-induced DSBs (147). In straightforward terms, this pathway involves simply ligating the two DNA ends back together. Due to the resection of DNA overhangs surrounding the DSB, NHEJ is sometimes considered the error-prone pathway of DNA DSB repair. NHEJ is active in all stages of the cell cycle, with activity peaking in G0 and G1 (12). The major proteins involved in NHEJ include the DNA-PKcs and the Ku70/80 heterodimer. Other core NHEJ proteins include artemis, XRCC4-XLF, and ligase IV. DNA-PKcs and Ku70/80 initially bind to the two ends of the DSB. The DNA ends are then processed by artemis, ligated by ligase IV and stabilized by XRCC4 and XLF.

**Figure 2:**
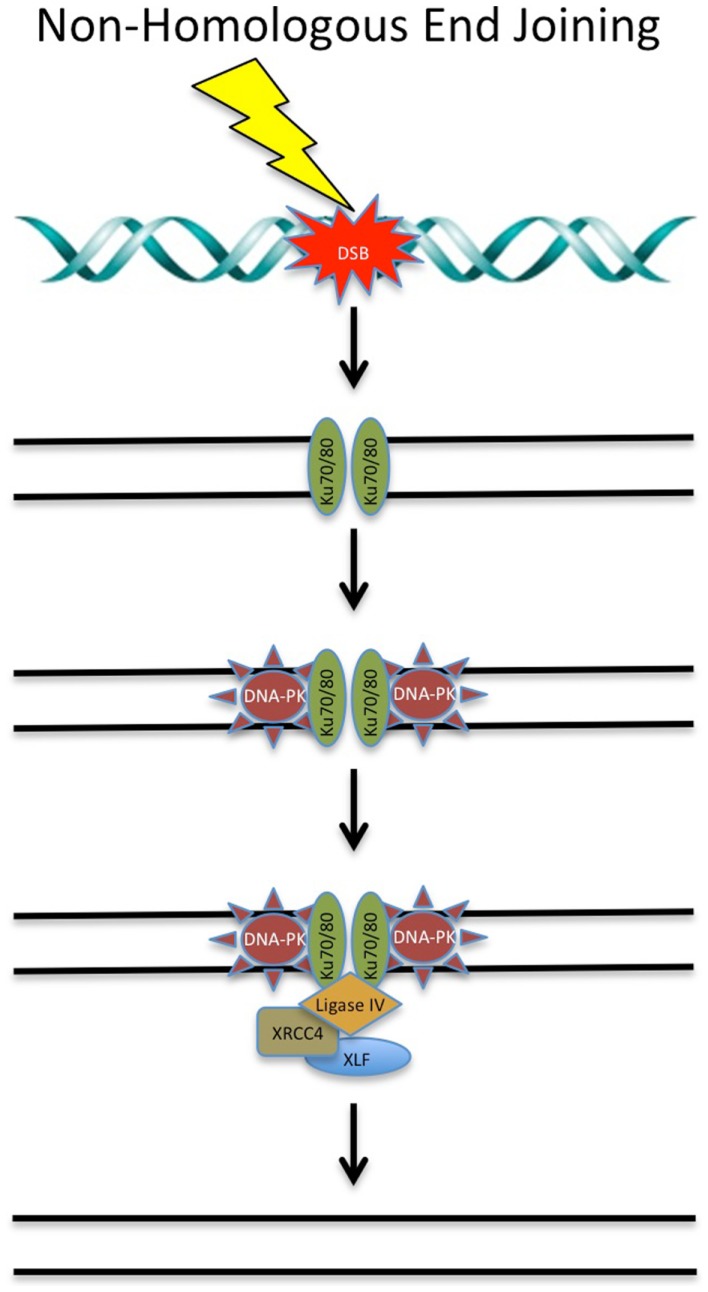
**DNA double-strand break repair via non-homologous end joining**. DSBs can be repaired via NHEJ throughout the cell cycle. Targeting of these proteins involved in NHEJ with chemotherapeutic compounds shows promise in the clinical setting. See text for details.

There are a number of key proteins involved in NHEJ that are targets of chemotherapeutic compounds (see Figure 2).

### DNA-dependent protein kinase catalytic subunit

The DNA-PK holoenzyme plays a major role in NHEJ and is involved in tethering the DNA ends at DSBs, allowing recruitment of other repair proteins. It also has serine/threonine kinase activity and can phosphorylate down-stream DNA repair proteins, leading to their activation. DNA-PKcs has been reported to be up-regulated in tumors or radiation-resistant cell lines, indicating that it is likely to have a role in tumor growth and survival (148, 149). In addition, B-cell chronic lymphocytic leukemia cells have been shown to escape apoptosis via the NHEJ pathway (150). Moreover, DNA-PKcs mutations have been detected in colorectal cancer cells (151). In light of the above and due to its pivotal role in NHEJ, DNA-PKcs is a protein of interest in developing new cancer treatments [reviewed in Ref. (152)].

Several inhibitors of DNA-PKcs have been identified, the most efficient of which target the ATP-binding pocket of the DNA-PKcs kinase domain (153). Compound library studies have identified several specific inhibitors of DNA-PKcs, but their development as cancer therapies has been restricted by weak pharmacokinetics.

Wortmannin, a metabolite of the fungus *Penicillium funiculosum* was one of the first DNA-PKcs inhibitors and has been widely used to study DNA-PKcs experimentally. This drug was used in the first studies that showed that inhibition of DNA-PKcs inhibited DNA DSB repair and enhanced the tumor-killing properties of agents that induce DNA damage, such as etoposide and IR. Although it can efficiently inhibit DNA-PKcs at an IC_50_ of 5 nM, its poor solubility, lack of specificity, and *in vivo* toxicity, have ensured that wortmannin has little clinical application (154).

As discussed earlier, another non-specific DNA-PKcs inhibitor utilized in several studies is LY294002, the morpholine derivative of the plant flavonoid quercetin. This inhibitor binds the kinase domain of DNA-PKcs with an IC_50_ of 1.4 μM (154). Like wortmannin, the clinical use of this inhibitor is limited by its lack of specificity and *in vivo* toxicity. In addition, LY294002 also displays rapid metabolic clearance in 1 h. Despite its limitations, LY294002 has proved useful as a foundation for biochemical modification, leading to several, more specific, clinically viable compounds (155).

An example of a compound developed from LY294002 is NU7026, which possesses 50-fold more selective inhibition of DNA-PKcs than other PI3Ks, with an IC_50_ of 0.23 μM against DNA-PKcs (156). This compound was found to enhance the tumor growth inhibition of several chemotherapy drugs, including daunorubicin, idarubicin, doxorubicin, and etoposide (157). However, due to metabolic instability, it is unlikely that high enough concentrations of NU7026 could be achieved in tumors to allow treatment in conjunction with chemotherapy or radiation treatment. Like other DNA-PK inhibitors, NU7026 also displays poor solubility in saline solutions (158).

Another DNA-PKcs inhibitor that resulted from the modification of LY294002 is NU7441, which strongly inhibits DNA-PKcs and has an IC_50_ of 0.3 μM. Treatment of cells with this drug led to an increase in HR and the persistence of IR- and doxorubicin-induced DSBs (159). Cellular treatment with NU7441 was also shown to delay the repair of IR- and etoposide-induced DSBs, in turn enhancing the tumor cell-killing properties of these treatments (160). In animal models, xenograft studies showed that NU7441 could increase the tumor growth inhibition of etoposide twofold with no increased toxicity. NU7441 has recently been shown to inhibit the multidrug-resistance 1 (MDR1) protein, in addition to DNA-PK, which may increase its therapeutic potential when combined with MDR substrates (161).

Two other inhibitors of DNA-PK have also been shown to sensitize cells to DNA-damaging agents, SU11752 and OK-1035 (162, 163). Unfortunately, both compounds displayed weak pharmacokinetic properties making them unsuitable for further clinical development.

Another agent, NK314 was already used in the clinic as a topoisomerase II alpha (TIIa) inhibitor and was also found to promote the degradation of DNA-PKcs, leading to defective DNA DSB repair. DNA-PKcs is highly expressed in adult T-cell leukemia–lymphoma (ATL), so NK314 may potentially be used as a dual targeting anticancer agent for treatment of ATL (164). A clinical trial for the use of NK314 in ATL patients is currently underway.

CC-115 is a DNA-PKcs inhibitor that is also undergoing early clinical evaluation. CC-115 is a dual inhibitor for DNA-PKcs and mTOR and the first clinical trial aims to assess its safety and action in patients with advanced solid tumors and hematologic malignancies that are unresponsive to standard therapies.

In summary, due to the role DNA-PKcs plays in DNA DSB repair via C-NHEJ and its overexpression in many cancers it was implicated as a suitable target for inhibition. Although several DNA-PKcs inhibitors have reached the pre-clinical evaluation stage, their use in patients have been limited by inadequate pharmacokinetics; as they are generally metabolically unstable, a high cellular concentration is unable to be achieved and therefore they are not clinically viable to potentiate other forms of cancer therapy. The use of antibody and oligonucleotide approaches to target DNA-PKcs may overcome the pharmacokinetic restrictions of small molecule inhibitors. However, there is still hope for this area of treatment as the crystallographic structure of DNA-PKcs was recently reported, allowing more efficient small molecule inhibitors of DNA-PKcs to be developed (165, 166). Computer-modeled compound design will allow targeting of the DNA-PK auto-phosphorylation sites or the DNA-PK/Ku80 interface, which are predicted to be more efficient than current DNA-PKcs inhibitors.

### Ku70/Ku80

The levels of the regulatory subunit of the DNA-PKcs holoenzyme, Ku70/80, like DNA-PKcs, are also increased in many tumors, which suggests that tumors may rely on Ku70/80 for survival (149). Indeed, it was shown that depletion of Ku70 or Ku80 using shRNA inhibited the growth of pancreatic tumor cells (167). Ku70- or 80-depletion also sensitized pancreatic cells to IR, suggesting that it may be a potential target for inhibition in cancer therapy in the future, although to date specific inhibitors have not been identified.

### DNA ligase IV

DNA ligase IV is an ATP-dependent DNA ligase that catalyzes the ligation step in NHEJ. Together with XRCC4 and XLF, DNA ligase IV forms a functional complex that is central to NHEJ (168–171). All DNA ligases catalyze the formation of the DNA phosphodiester bond in a three-step process. The initial hydrolysis of ATP leads to the covalent linkage of an AMP moiety to a specific lysine residue within the active site of DNA ligase and the subsequent release of pyrophosphate. A DNA adenylate intermediate is formed through the transfer of the AMP moiety from the adenylated ligase to the 5′ terminus of a DNA nick with a 5′ phosphate and 3′ hydroxyl terminus. Finally, the non-adenylated DNA ligase interacts with the DNA adenylate and the termini are linked together via a phosphodiester bond, with the final release of AMP (172).

Inhibiting the activity of DNA ligase IV has become an attractive approach to increase the sensitization of cancer cells to DNA damage. As DNA ligation is required during DNA repair and replication, cells deficient in DNA ligases have been shown to be sensitive to a variety of DNA-damaging agents (173). To date, there are two described DNA ligase IV inhibitors, L189 and SCR7.

A computer-aided drug design approach, based on the structure of human DNA ligase I complexed with nicked DNA, was performed to identify low molecular weight inhibitors of DNA ligases that specifically abrogate functional interactions between the ligase and nicked DNA (174). L189 was 1 of a 192 potential candidate inhibitors chosen from this rational approach. L189 was further characterized *in vitro*, and shown to inhibit DNA ligase I, III, and IV in DNA joining assays using purified protein and in DNA replication, BER, and NHEJ in cell extract assays. Specifically, L189 inhibited the ligase reaction by >90%, however, only had a minimal effect on T4 DNA ligase. In cell culture, L189 was found to be cytotoxic, using colony-forming assays. Furthermore, L189 significantly increased the cytotoxicity of the DNA-damaging agents MMS and IR in three cancer cell lines (breast, cervical, and colon) but not in a normal breast epithelial cell line. Hence, *in vitro* data suggest that L189 is a potential lead compound for the development of chemotherapeutics (174). However, *in vivo* data and subsequent clinical trials are required to further substantiate these results.

SCR7 is a L189 derivative that was identified by an *in silico* docking approach, as a specific inhibitor of DNA ligase IV. SCR7 disrupts the sealing of DSBs by ligase IV by interfering with its binding to DNA. *In vitro*, SCR7 inhibits NHEJ in a ligase IV-dependent manner, leading to the accumulation of DSBs and subsequent cytotoxicity. SCR7 was used on four different mouse models to determine tumor progression. Three of the four mouse models were responsive and SCR7 was found to significantly reduce tumor progression and increase lifespan, relative to the control. SCR7 slowed the progression of the tumor by activating the p53-mediated apoptotic pathway and hence increasing lifespan. Additionally, when SCR7 was co-administered with IR and etoposide in mouse models, it significantly increased the sensitivity of tumors (175). This study demonstrates that inhibitors of DNA repair, in combination with existing chemo and radiotherapy, may lead to a better efficacy of treatment.

### XRCC4

The initial step in NHEJ is the recognition and binding of the Ku70/80 heterodimer to the DSB (176). After Ku70/80 is bound to DSB ends, it recruits other NHEJ factors such as XRCC4 to the site of damage (177). Ku70 and XRCC4 directly interact with each other and XRCC4 may act as a flexible tether between Ku70/80 and DNA ligase IV (176). XRCC4 has no known enzymatic activity, but may function as an additional NHEJ scaffolding protein, responsible for the recruitment of other NHEJ factors to the site of the damage (177).

In mice, XRCC4 deficiency has been shown to cause late embryonic lethality (178) and mouse Xrcc4 was found to stimulate adenylation of DNA ligase IV *in vitro*, the first chemical step in DNA ligation (179).

Since XRCC4 plays a central role in the repair of DSB by NHEJ (177), the presence of active XRCC4 in cells may decrease DSB-mediated apoptosis in cancer cells during radiotherapy. Therefore, the use of potent XRCC4 inhibitors has the potential to enhance radiotherapy outcomes in patients.

Salvianolic acid B, lithospermic acid, and 2-*O*-feruloyl tartaric acid were identified as potent agents for interrupting XRCC4-mediated DNA repair, from a screen involving 20,000 compounds from the traditional Chinese medicine (TCM) database (180). The compounds were modeled for their binding affinities to the DNA ligase IV binding region on XRCC4 and for all three inhibitors, the protein–ligand interactions were focused at Lys188 on chain A and Lys187 on chain B of XRCC4. From this study, salvianolic acid B, lithospermic acid, and 2-*O*-feruloyl tartaric acid are potential enhancers of radiotherapy and furthermore, may have characterized the key binding elements for inhibiting XRCC4 activity (180). While this study is promising, the efficacy of these inhibitors has yet to be tested using *in vitro* and *in vivo* models.

Inhibiting the XRCC4/DNA ligase IV complex formation could also provide a novel strategy for inhibiting NHEJ. The minimal inhibitory fragment of the XRCC4-interacting region (XIR) capable of abolishing XRCC4/XIR complex was recently identified (181). The key interfaces of ligase IV necessary for interaction with XRCC4 were identified by the development a competitive displacement assay using ESI-MS/MS. The results suggest that by targeting the interface of helix 2 of DNA ligase IV, modulators that inhibit the XRCC4/DNA ligase IV complex may be identified. In addition, adjuvant compounds to further block the XRCC4/DNA ligase IV complex may be discovered by further targeting helix 1 and the loop regions of the helix–loop–helix clamp, which offer a secondary target surface (181). While this study has the potential to identify inhibitors of XRCC4, to date, inhibitors that have been tested *in vivo* and in clinical trials have not been described.

### XCRCC4-like factor

XCRCC4-like factor/cernunnos (XLF/cer) is a recently discovered XRCC4 interaction partner. XLF directly interacts with the XRCC4-ligase IV complex both *in vitro* and *in vivo*. Furthermore, siRNA knockdown of XLF in mammalian cells gives rise to radiosensitivity and impaired NHEJ and the re-introduction of wild-type XLF into defective cells corrects the observed defects (171). Data suggest that following DSBs, XLF accumulates at DNA damage sites via constitutive interaction of the XRCC4 head domains and XLF globular head domains in the XRCC4–DNA ligase IV complex and dependent components of the DNA-PK complex. Following this, XLF stimulates the ligation of complementary and non-complementary DNA ends via XRCC4 and DNA ligase IV. XLF in summary ensures the accuracy of the joining of DSBs during NHEJ and V(D)J recombination (182).

While there are no inhibitors of XLF in current use, inhibitors that abrogate the formation of the XRCC4/XLF/DNA ligase IV functional complex that is central to NHEJ may provide a novel strategy to improve radiotherapy outcomes in patients.

### p53-binding protein 1

p53-binding protein 1 (53BP1) is a human BRCT protein that was initially identified by a yeast 2-hybrid screen as a p53-interacting protein (183). 53BP1 binds to p53 and enhances p53-mediated transcriptional activation. 53BP1 is a central regulator of DNA DSB repair and functions to promote the end joining of distal DNA ends induced during V(D)J and class switch recombination. Additionally, 53BP1 is involved in the fusion of unprotected telomeres (184, 185). 53BP1 is an ATM substrate that forms nuclear foci in response to DNA damage (186) and promotes NHEJ while preventing HR. Recent evidence suggests that 53BP1 recruitment requires the direct recognition of a DSB-specific histone code and the choice of NHEJ vs. HR is dependent on BRCA1 (185).

The identification of specific chemotherapeutic compounds targeting 53BP1 and thereby sensitizing cancer cells to radiotherapy is an approach that requires further investigation.

### Alternative NHEJ

Recent studies have identified another DSB repair pathway, termed alternative NHEJ (A-NHEJ). This pathway comprises another simple end joining process that is normally suppressed by the C-NHEJ pathway and only operates when C-NHEJ and HR pathways are compromised. Therefore, A-NHEJ is generally considered a backup repair pathway and is implicated to be highly error-prone (187). It has been suggested that A-NHEJ may actually be comprised of several pathways due to the functional diversity of the A-NHEJ proteins identified so far. However, it has also been suggested that A-NHEJ results from the initiation and failure of C-NHEJ or HR, resulting in C-NHEJ or HR proteins already being present at the DSB. When the initiation of A-NHEJ follows unsuccessful C-NHEJ, C-NHEJ factors are already at the DSB, but instead of DNA ligase IV performing the ligation step, this is performed by DNA ligase 3 or 1 (188–190). It has also been suggested that A-NHEJ may also function to join DNA ends that have been processed by HR factors such as the MRN complex, CTIP, and BRCA1 (190–192). The A-NHEJ pathway has been implicated as enabling tumor cells that have disrupted HR or C-NHEJ pathways to survive, making it an attractive target for inhibition.

### DNA ligase 3α

A recent study demonstrated that KRAS mutated leukemic cells have increased levels of components of the A-NHEJ pathway, including DNA ligase 3α, PARP1, and XRCC1 and that these cells rely on the A-NHEJ for survival (193). In addition, it was also shown that depletion of DNA ligase 3α using RNAi sensitized the KRAS-mutant leukemic cells to chemotherapy. This suggests that targeting the A-NHEJ pathway may be a promising avenue for inducing synthetic lethality in combination with DNA-damaging agents in cells bearing KRAS mutations, for which there is currently no reliable treatment.

A list of all chemotherapeutic compounds targeting the NHEJ pathway is provided at Table 2.

**Table 2:** **Chemotherapeutic compounds targeting the non-homologous end joining DNA repair pathway**.

Compound	Class	Clinical phase	Combination
Wortmannin	DNA-PKcs and other PIKKs	–	–
LY294002	DNA-PKcs	–	–
NU7026	DNA-PKcs	–	–
NU7441	DNA-PKcs	–	–
SU11752	DNA-PKcs	–	–
OK-1035	DNA-PKcs	–	–
NK314	DNA-PKcs and topoisomerase II alpha	I	Monotherapy
CC-115	DNA-PKcs and mTOR	I	Monotherapy
L189	DNA ligase IV	–	–
SCR7	DNA ligase IV	–	–

## Conclusion

Human solid tumors have frequently been found to have pronounced genetic and gene expression heterogeneity, of both cancerous and the normal cells within the tumor. This diversity of cell populations within the tumor may explain why cancer is so resistant to therapy, including more targeted therapeutic approaches. The increased proliferation of cancer cells also places stress on the genome, with the fastest growing cell populations having an advantage in the environment. To increase growth rates and remove normal restrictions on growth, cancer cells evolve to have defects in the DNA repair pathways and in checkpoint signaling and apoptosis. As a result of these defects and increased metabolic activity, cancer cells are genomically unstable with the most aggressive cancers showing the most genetic instability. However, this instability differentiates the cancer cells from normal cells, potentially opening up therapeutic windows. The DSB repair pathway is the most promising of these therapeutic windows, as defects in this pathway are commonly associated with diseases such as cancer.

The disruption of the DSB repair mechanisms HR and NHEJ, via chemotherapeutic compounds used as either a monotherapy or in conjunction with radiotherapy, has shown promise in the clinical setting for the treatment of various cancers. The targeting of these processes can be further exploited as further investigations into the HR and NHEJ pathway lead to the identification of new potential targets. However, complete inhibition of HR and NHEJ for any extended time period is likely to be lethal to all dividing cells, therefore targeted or temporary inhibition is likely to be useful in conjunction with radiotherapy. Also, complete inhibition of NHEJ may lead to further genomic instability in normal cells, as it is the only pathway for repairing DSBs in non-dividing cells.

Further investigation into synthetic lethality, beyond the identified PARP/BRCA lethality, may lead to additional avenues to be targeted and exploited by the use of chemotherapeutic compounds. Promising targets to expand on PARP inhibition using synthetic lethality are other proteins involved in the HR pathway, including cancers with mutations in ATM, p53, Chk2, Rad51, and NBS1. Inhibition of other proteins involved in the DDR response has also shown promise when combined with BRCA mutations. *In vitro* depletion of Rad52 in BRCA2-deficient cells showed synthetic lethality when compared with BRCA2-competent cells (194). Recently, synthetic-lethal relationships in chromatin-regulating genes have been identified, including chromatin remodeling factors (195) and methyltransferases (196). The concept of synthetic lethality could allow the exploitation of differences between tumor cells and normal cells that have previously been considered to be intractable and it has been shown to be a promising means of selectively killing tumor cells.

Whilst there have been a number of good and promising results using chemotherapeutic compounds, there have been a number of failed studies. Ensuring that there are sufficient investigations completed both *in vitro* and *in vivo* confirming the specificity and pharmacokinetics of these chemotherapeutic compounds before introduction in the clinical setting is critical. As the response to chemotherapeutic compounds becomes more predictable and with the identification of specific tumor biomarkers, this will allow for targeted, more efficient cancer treatments. Inhibition of these DSB repair proteins holds great promise for the future of cancer therapy in the future.

## Author Contributions

All authors were involved in the conception of the manuscript, the drafting and/or critically reviewing of the manuscript, have approved the final version for publishing, and have agreed to be accountable for the work.

## Conflict of Interest Statement

The authors declare that the research was conducted in the absence of any commercial or financial relationships that could be construed as a potential conflict of interest.
